# Association mapping for *Striga* resistance and agronomic‐related traits in sorghum

**DOI:** 10.1002/tpg2.70129

**Published:** 2025-10-05

**Authors:** Wilbert T. Mutezo, Moosa M. Sedibe, Justice Norvienyeku, Bingting Lai

**Affiliations:** ^1^ Department of Agriculture Central University of Technology Bloemfontein South Africa; ^2^ School of Tropical Agriculture, Health and Forestry Hainan University College, Danzhou Campus of Hainan University Danzhou China

## Abstract

Over 50% of arable land available for cereal production in sub‐Saharan Africa is severely infested with *Striga hermonthica* (Del.) Benth, posing a significant challenge to agricultural productivity in the region. In this study, we performed association mapping of plant height, panicle height, number of leaves per plant, field fresh grain weight, dry grain weight, and chlorophyll with 6,094,317 single nucleotide polymorphism (SNP) markers for *Striga* resistance genes in diverse sorghum [*Sorghum bicolor* (L.) Moench] breeding lines and varieties released for resistance breeding. Chromosomes containing significant SNPs in FASTmrMLM and FarmCPU models were identified and computed. Chromosomes 1, 2, 3, 4, and 6 harbored SNPs significant for *Striga* tolerance in sorghum for agronomic‐related traits. Agronomic traits measured revealed significant SNP counts as follows: plant height (4), panicle height (3), leaves per plant (2), foliar fresh grain weight (8), dry grain weight (2), and chlorophyll content (3). After successful validation, the 22 newly identified SNP markers linked to *Striga* resistance can be used for trait introgression and marker‐assisted selection to increase *Striga* resistance in sorghum. We detected 12 SNPs using the FASTmrMLM model without adjusting the threshold level. However, no significant SNPs were detected with FarmCPU before the threshold was adjusted. Also, we identified 95 significant SNPs upon lowering the Bonferroni threshold value to *p *< 0.001. The parent materials for the intraspecific cross that produced the currently accessible molecular map were selected from the gene pool of cultivated sorghum. This map is invaluable for real‐world breeding applications. Subsequent crosses among cultivated sorghum genotypes of interest to breeders will likely produce polymorphic segregating Diversity Array Technology (DArTSeq) markers within the cultivated gene pool.

## Core Ideas

- Twenty‐two single nucleotide polymorphisms (SNPs) have been identified for *Striga* resistance.
- Chromosomes 1, 2, 3, 4, and 6 harbor SNPs of *Striga* tolerance in sorghum.
- *Striga* resistance is a very important trait for sub‐Saharan Africa in cereal crops.

## Plain Language Summary

Over 50% of arable lands available for cereal production in sub‐Saharan Africa are severely infested with *Striga* spp., posing a significant challenge to agricultural productivity in the region. In this study, we performed association mapping of plant height, panicle height, number of leaves per plant, field fresh grain weight, dry grain weight, and chlorophyll with 6,094,317 single‐nucleotide polymorphism (SNP) markers for *Striga* resistance genes in diverse sorghum breeding lines and varieties released for resistance breeding. Chromosomes containing significant SNPs in FASTmrMLM and FarmCPU models were identified and computed. Chromosomes 1, 2, 3, 4, and 6 harbored SNPs significant for *Striga* tolerance in sorghum for agronomic‐related traits. Agronomic traits measured revealed significant SNP counts as follows: plant height (4), panicle height (3), leaves per plant (2), field fresh grain weight (8), dry grain weight (2), and chlorophyll content (3). After successful validation, these can be used in future breeding programs for marker‐assisted selection.

## 1 INTRODUCTION

Parasitism by *Striga* spp. is one of the most significant threats to efforts to increase agricultural productivity in sub‐Saharan Africa. *Striga* spp. are endemic to vast proportions of arable lands available for cereal cultivation in sub‐Saharan Africa (Abdullahi & Musa, 2024; Yonli et al., 2024). Numerous biotic variables, such as parasitic weeds, infectious diseases, viruses, and insect pests, pose hazards to sorghum crops. Diseases caused, particularly by biotic factors, inflict significant financial losses on sorghum production yearly (Aslam et al., 2024). *Striga* spp. is among the predominant invasive and pervasive weeds that limit sorghum productivity worldwide, especially in Africa. In severely infested soils, *Striga* spp. can cause up to 100% yield losses (Rouamba et al., 2024). Overall, deploying contemporary genetic modification techniques to facilitate the identification and cloning of candidate genes associated with essential agronomic traits, including high yield and biotic stress resistance, will enhance sorghum yield and resistance performance (Mukherjee et al., 2024).

Over the past 25 years, *Striga* resistance research in sorghum has progressed from conventional breeding to advanced genomics, reflecting a deep and multidimensional scientific effort. Early work focused on identifying resistant genotypes and understanding host–parasite interactions under field and controlled environments (Ejeta & Butler, 1993; Haussmann et al., 2000, 2004; Ramaiah, 1983). Marker‐assisted selection (MAS) in the early 2000s enabled the mapping of quantitative trait loci (QTLs) associated with resistance traits such as low emergence, delayed attachment, and host hypersensitivity (Amusan et al., 2008; Haussmann et al., 2004; Haussmann & Hess, 2001). Physiological studies also revealed key pre‐attachment resistance mechanisms, particularly reduced strigolactone exudation, a critical germination stimulant for *Striga* (Gobena et al., 2017; Yoneyama et al., 2010).

Comparative research in maize (*Zea may* L.), pearl millet (*Pennisetum glaucum* (L.) R. Br.), and rice (*Oryza sativa* L.) has similarly identified QTLs and resistance mechanisms, underscoring the conserved genetic architecture of *Striga* resistance across cereals (Ali et al., 2016; Gurney et al., 2006; Kountche et al., 2019; Rezig et al., 2016). For instance, resistance in maize has been associated with low production of germination stimulants and host cell wall reinforcement (Amusan et al., 2008; Badu‐Apraku et al., 2016), while rice studies have demonstrated natural variation in *Striga*‐responsive strigolactones and transporter genes (Cardoso et al., 2014; Jamil et al., 2018).

In sorghum, the advent of genome‐wide association studies (GWAS) has enabled fine mapping of resistance loci, uncovering candidate genes involved in strigolactone biosynthesis (e.g., *LGS1*) and post‐attachment responses such as hypersensitive reaction and xylem blocking (Mohamed et al., 2003; Muchira, 2022; Mutinda et al., 2023; Mwangangi et al., 2025). Pan‐genomic, transcriptomic, and metabolomic approaches are further enriching our understanding of resistance diversity and gene expression networks (Earecho & Nebiyu, 2025; Greenhill, 2023; Maulana et al., 2023). Collectively, these studies reflect a robust and integrated research trajectory aimed at developing durable *Striga*‐resistant varieties across cereal crops through molecular breeding and precision agriculture.

Genetic wide association examines the whole genome for correlations between behavioral changes and genetic markers, typically single nucleotide polymorphisms (SNPs) (Abdellaoui et al., 2023; Beck et al., 2023; Wu et al., 2023). The primary objective of a GWAS is to distinguish spurious relationships that may arise from confounding factors, such as kinship and population structure, from genuine marker‐trait connections resulting from actual genetic effects (Beck et al., 2023). Previous studies have shown that selecting a suitable and optimum performance of GWAS models to support GWAS‐based analysis requires an extensive investigation (Wu et al., 2023). GWAS have effectively identified genetic areas linked to traits of significant agronomic interest in sorghum (L. Zhang, Xu, et al., 2023; Y. Zhang, Fan, et al., 2023). Molecular markers linked to desirable qualities are crucial for identifying genetic loci or alleles that cause phenotypic variations based on linkage disequilibrium (Lee et al., 2023).

This study aimed to uncover SNP‐based genomic associations between *Striga* resistance and key agronomic traits—such as plant height, grain weight, and chlorophyll content in diverse sorghum germplasm.

## 2 MATERIALS AND METHODS

### 2.1 Phenotype data

A total of 74 sorghum genotypes from breeding lines and released varieties were collected in South Africa and Zimbabwe (Table 1). Of these, 20 genotypes were obtained from the Agricultural Research Council – Grain Crops in South Africa, and 54 were from Seedco, Rattary Arnold Research Station, Zimbabwe. Seeds were grown under favorable conditions in a greenhouse.

**TABLE 1:** List of sorghum genotypes obtained from South Africa and Zimbabwe.

No.	Genotype code	Source	No.	Genotype code	Source	No.	Genotype code	Source
1	SCSHYB012150	Zimbabwe	26	SCSHYB017081	Zimbabwe	51	SCSHYB015027	Zimbabwe
2	SCSHYB013103	Zimbabwe	27	SCSHYB017139	Zimbabwe	52	SCSHYB015028	Zimbabwe
3	SC SILA	Zimbabwe	28	SCSHYB017166	Zimbabwe	53	SCSHYB017082	Zimbabwe
4	SCSHYB01150	Zimbabwe	29	SCSHYB017165	Zimbabwe	54	SA2133	South Africa
5	SA1595	Zimbabwe	30	SCSHYB017086	Zimbabwe	55	SA1617	South Africa
6	CHR19	Zimbabwe	31	CHR 25	Zimbabwe	56	SA1939	South Africa
7	MACIA	Zimbabwe	32	SCSHYB012101	Zimbabwe	57	SA1800	South Africa
8	SC SMILE	Zimbabwe	33	SCSHYB017075	Zimbabwe	58	SA2527	South Africa
9	SCSHYB011120	Zimbabwe	34	SCSHYB012116	Zimbabwe	59	SA1796	South Africa
10	CHR 28	Zimbabwe	35	SCSHYB012112	Zimbabwe	60	SA2490	South Africa
11	SA1600	South Africa	36	SCSHYB015002	Zimbabwe	61	SA3280	South Africa
12	CHR 20	Zimbabwe	37	SCSHYB017090	Zimbabwe	62	SA1873	South Africa
13	SA1596	South Africa	38	SCSHYB017084	Zimbabwe	63	SA3100	South Africa
14	CHR22	Zimbabwe	39	SCSHYB017085	Zimbabwe	64	SA1618	South Africa
15	SCSHYB017089	Zimbabwe	40	SCSHYB017083	Zimbabwe	65	SA2538	South Africa
16	SCSHYB017087	Zimbabwe	41	SCSHYB015017	Zimbabwe	66	SA3028	South Africa
17	SCSHYB012108	Zimbabwe	42	SCSHYB015018	Zimbabwe	67	SA2362	South Africa
18	SCSHYB017088	Zimbabwe	43	SCSHYB015019	Zimbabwe	68	SA2548	South Africa
19	SCSHYB012121	Zimbabwe	44	SCSHYB015020	Zimbabwe	69	SA2311	South Africa
20	SCSHYB012156	Zimbabwe	45	SCSHYB015021	Zimbabwe	70	SA2475	South Africa
21	SCSHYB012120	Zimbabwe	46	SCSHYB015022	Zimbabwe	71	SA2070	South Africa
22	SC SILA(KRC) 0P45	Zimbabwe	47	SCSHYB015023	Zimbabwe	72	SA4186	South Africa
23	SCSHYB012157	Zimbabwe	48	SCSHYB015024	Zimbabwe	73	SA1794	South Africa
24	SCSHYB012155	Zimbabwe	49	SCSHYB015025	Zimbabwe	74	SA2093	South Africa
25	SCSHYB012160	Zimbabwe	50	SCSHYB015026	Zimbabwe			

All the genotype populations were grown under greenhouse and glasshouse conditions. The sorghum genotypes were planted in pots with topsoil mixed with compost (3:1) at the Rattary Arnold Research Station in March 2022. A triplicate 3 × 25 alpha‐lattice design was used for the experiments. After screening for TG resistance, mature panicles were harvested and dried for storage (<15% moisture).

### 2.2 *Striga* and agronomic attributes

#### 2.2.1 Planting procedure

Three levels of *Striga hermonthica* (0, 2.5, and 5 mg) were tested in pots to evaluate their effect on the agronomic parameters of various sorghum genotypes. Ferruginous soil from an area free of *S. hermonthica* was used to fill plastic pots 40 cm in diameter with three pots of disinfected sorghum seeds, which were then promptly irrigated. About 15 g of compound D (7:14:7) fertilizer was added to each pot and properly mixed with the soil. About 2.5 and 5 mg of *S. hermonthica* seeds were placed in the top 6 cm of soil in each pot, respectively, to accomplish *S. hermonthica* inoculation. The pots were watered every day to prevent moisture deficit. After emergence, the plants were thinned to two plants per pot. Weeds other than *S. hermonthica* were regularly handpicked.

**Core Ideas**

- Twenty‐two single nucleotide polymorphisms (SNPs) have been identified for *Striga* resistance.
- Chromosomes 1, 2, 3, 4, and 6 harbor SNPs of *Striga* tolerance in sorghum.
- *Striga* resistance is a very important trait for sub‐Saharan Africa in cereal crops.

#### 2.2.2 Experimental design

Experimental pots were laid out in a split‐plot design with three *S. hermonthica* levels (0, 2.5, and 5 mg/pot) as the main factor and 74 cultivars (sub‐factor), and with two replications.

#### 2.2.3 Measurements

Data were gathered from both plants in each pot. For comparison, an uninfected control of *S. hermonthica* was included. A handheld chlorophyll meter was used to measure the chlorophyll contents on week 3 following emergence to week 10. Without causing any harm to the plant material, the chlorophyll concentration from undamaged leaf samples was immediately measured and shown using the MC‐100 chlorometer. Using values of µmol of chlorophyll per meter square, the meter was calibrated to measure the concentration of chlorophyll. The height and quantity of leaves of sorghum were measured at 4, 5, 6, 7, 8, 9, and 10 weeks after sowing. Measurements were collected on foliar fresh weight, length, and grain weight of sorghum panicles as shown in Table S1.

Sand and topsoil (1:3) were cleaned 14 days before planting. At the Rattary Arnold Research Station in Harare, Zimbabwe, seeds of various sorghum genotypes were sown in 20‐cm diameter pots filled with a 1:3 mixture of topsoil and sand in a greenhouse. Young, succulent, and fresh leaves were collected from each genotype at the five‐leaf stage and oven‐dried for 24 h at 35°C. For sequencing and SNP analysis, the leaf samples were placed in a resealable bag, labeled with the relevant genotypic code, and delivered to the Seqart Africa, Hosted Institution at the International Livestock Research Institute (ILRI), Old Naivasha Road, Nairobi, Kenya. The genetic diversity of the 75 sorghum genotypes was analyzed using DArTSeq.

DArTseq (Diversity Arrays Technology Pty Ltd., Canberra, Australia) was used for DNA extraction and sequencing (Mudaki et al., 2023). Approximately 1 g of young leaf tissue from each genotype was used for genomic DNA extraction. Genomic DNA was extracted from the oven‐dried leaves using a modified cetyltrimethylammonium bromide (CTAB)/chloroform/isoamyl alcohol technique (Doyle, 1991). The dry leaf tissue was ground and combined with 2% pre‐warmed (60°C) CTAB isolation buffer (Sigma, Saint Louis, USA) containing 1.4 M NaCl, 100 mM Tris (pH 8.0), and 20 mM EDTA. Afterward, the mixture was placed into a 2‐mL microcentrifuge tube and allowed to sit at 60°C for 1 h. Chloroform‐isoamyl alcohol (24:1) (Sigma, St. Louis, MO, USA) was used to extract DNA, and two volumes of isopropanol were used to precipitate the extract. After rinsing with 70% EtOH, the pellet was dried and dissolved in 100 µL of TE buffer containing 50 µg/mL of RNase A (Sigma, Saint Louis, USA). Using 0.8% agarose gel electrophoresis, the isolated DNA was measured and normalized to 50 ng/µL for SNP genotyping and DArTseq.

### 2.3 Diversity array technology analysis

The DNA was processed through digestion/ligation procedures by substituting two separate adaptors compatible with *PstI* and *SphI* with a single *PstI‐*compatible adaptor (Kilian et al., 2012). An Illumina flow cell attachment sequence with staggered sequences of barcode regions of different lengths, similar to that published by Elshire et al. (2011), was used to construct a PstI‐compatible adapter (Elshire et al., 2011). A flow cell‐attachment area with an overhang sequence compatible with *SphI* was present in the reverse adapter. In 30 rounds of polymerase chain reaction (PCR), only “mixed fragments” (*PstI*–*SphI*) were successfully amplified. The reaction conditions were as follows: 94°C for 1 min, followed by 30 cycles of 94°C for 20 s, 58°C for 30 s, 72°C for 45 s, and 72°C for 7 min. Subsequently, each sample from the 96‐well microtiter plate was amplified at equimolar concentrations, bulked, and subjected to Bot (Illumina) bridge PCR. Sequencing was performed using the Illumina HiSeq 2500 system (Illumina, San Diego, CA, USA). Seventy‐seven rounds of single‐read sequencing were performed.

Proprietary DArT analytical pipelines (PLs) were used to process the sequences generated from each lane. For the primary pipeline analysis, low‐quality sequence fragments with reproducibility below 90% and read depths lower than 3.5 for SNPs or 5 for presence‐absence markers were filtered out. The barcode region was subjected to more stringent selection criteria: the sequences were assigned to specific samples carried within the barcode splitting step with high reliability, and no samples were dropped due to low coverage across loci. Individual sequences failing to meet the above criteria were removed. For marker calling, almost 2.5 million sequences were found for each barcode and sample. For reference alleles and SNP alleles, the average browsing depth across loci was 9.2 reads per individual and 6.5 reads for each locus. Lastly, identical sequences were compiled into files called “fastqcoll.” Using collapsed tags with many members as a template, DArT PL's proprietary technique was used to process the fastqcoll files. This approach corrects low‐quality bases by using singleton tags on correct bases.

Using DArTsoft14 (Diversity Arrays Technology Pty Ltd., Canberra, Australia), the secondary pipeline for PL's proprietary SNP, and SilicoDArT (presence/absence of restriction fragments in representation; PA markers) calling algorithms made use of the processed fastqcoll files. Using the C++ algorithm software developed by DArT PL, SNP calling was performed for every tag from every library included in the DArTsoft14 analysis and clustered at a three‐blink distance. The balance of read counts for the allelic pairing method was used to parse the clusters into distinct SNP loci. The algorithm program was subsequently enhanced with further selection criteria, with the help of an examination of approximately 1000 controlled cross‐populations. To simplify the selection of technical parameters needed to distinguish genuine allelic variants from paralogous sequences, the populations were evaluated against the Hardy–Weinberg equilibrium of alleles to determine the presence of variations. Furthermore, several samples were processed from DNA to allelic calls as technical duplicates. The primary criterion for selecting high‐quality/low‐error‐rate markers was scoring consistency. A high average browsing depth per locus (>30 reads/locus across all markers) guarantees calling quality. About 50 ng/µL of DNA was used for the GBS analysis. Metadata of 74 sorghum genotypes form South Africa and Zimbabwe from SNP analysis using Dartseq and Dartseq SNP analysis raw data for 74 sorghum genotypes are shown in Tables S2 and S3, respectively.

## 3 DATA ANALYSIS

### 3.1 Agronomic attributes and *Striga* tolerance

The data were subjected to descriptive statistics and analysis of variance (ANOVA) using the computer program SAS (XLSTAT 2020.5.1.1046). For the statistical analysis, one‐way ANOVA was used. Experimental means were separated using the least significant differences (5% level). Pearson's correlation coefficient for all variables was also determined.

### 3.2 Population structure

SNP density plots were analyzed and plotted using *rMVP* package. The population structure was determined using the LEA package. The optimum number of clusters/subpopulations (K) was analyzed using the *snmf* function and visualized using the cross‐entropy criterion of the LEA package. A Q matrix was developed and used to plot admixture proportions.

### 3.3 Association analysis

Phenotypic traits with an SD of 0 were excluded from GWAS. GWAS was performed using the FarmCPU model in the GAPIT package. Furthermore, a multi‐locus random GWAS analysis was performed on agronomic traits using the FASTmrMLM model in the mrMLM package to identify significant SNPs without adjusting the threshold. Manhattan and QQ plots were generated using *the qqman* package.

## 4 RESULTS

### 4.1 Analysis of variance and correlation analysis for agronomic traits of sorghum exposed to *Striga*

Table 2 shows that plant height had a moderate mean of 111.48 cm with a coefficient of variation (CV) of 20.21%, indicating moderate variability across genotypes. The number of leaves was stable with a low CV of 13.9% and a mean of 9.19. In contrast, grain weight and fresh weight had high variability, with CVs of 72.25% and 68.08% and means of 51.68 and 70.34 g, respectively, suggesting strong differences in yield under Striga stress. The Striga germination score showed extreme variability (CV = 289.33%), ranging from 1 to 162, indicating highly diverse levels of resistance among genotypes.

**TABLE 2:** Summary statistics of agronomic attributes of sorghum exposed to *Striga*.

Parameter	Mean	SD	Min	Max	CV
Plant height	111.48	22.53	18	165	20.21
Chlorophyl	20.09	8.5	−27.8	50.5	42.3
Number of leaves	9.19	1.28	5	12	13.9
Fresh weight	70.34	47.89	5	238	68.08
Grain weight	51.68	37.34	3	181	72.25
Panicle height	22.11	4.61	6	33	20.84
*Striga* germination score	2.64	7.65	1	162	289.33

Table 3 indicates that treatment had a highly significant effect (*F* = 595.63, *p* < 2.2e‐16), confirming the strong impact of *Striga* on plant performance. Variety also had a significant effect (*F* = 9.37, *p* < 2.2e‐16), showing genetic variability. The interaction between variety and treatment was significant (*F* = 3.51, *p* = 1.38e‐14), indicating differential genotypic responses to *Striga*. Replication had no significant effect (*F* = 0, *p* = 1).

**TABLE 3:** Analysis of variance of 47 sorghum genotypes exposed to *Striga*.

	DF	SS	MS	*F*‐value	*p*r (>F)	
Replication	1	0.0000	0.000	0.0000	1	
Variety	73	321.78	4.408	9.3655	<2.2e‐16	***
Treatment	1	280.33	280.33	595.626	<2.2e‐16	***
Variety: Treatment	73	120.67	1.653	3.5121	1.38E‐14	***
Residuals	301	141.67	0.471			

*** *p *< 0.001, ** *p *< 0.01, * *p *< 0.05.

**FIGURE 1:**
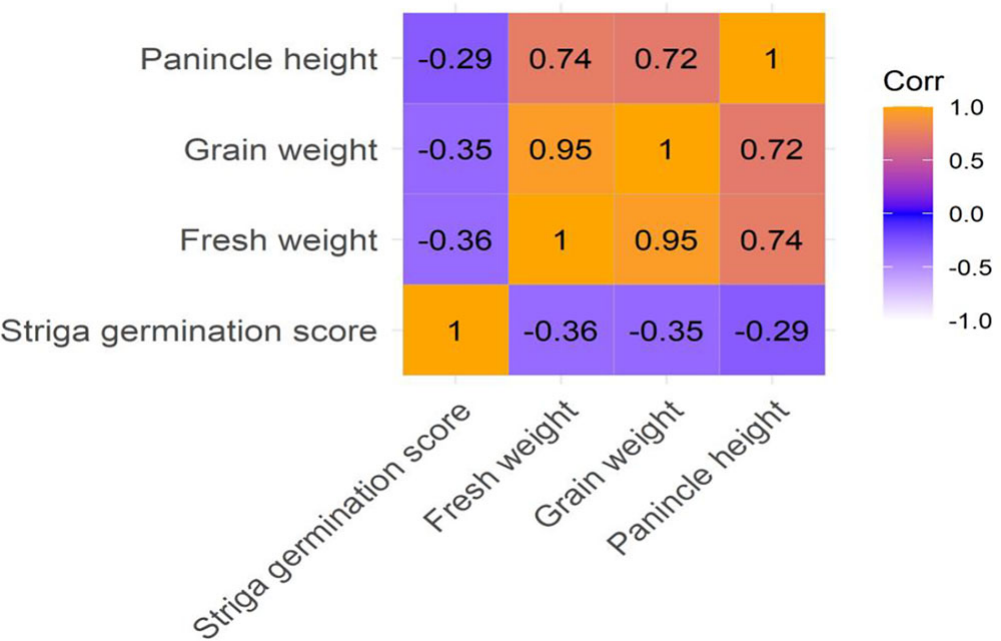
Correlation matrix of agronomic attributes of 74 sorghum genotypes exposed to *Striga*.

Figure 1 shows the correlation matrix for agronomic traits under *Striga* stress. Strong positive correlations were observed between plant height and fresh weight (*r* = 0.72) and between fresh weight and grain weight (*r* = 0.85), suggesting that taller plants and those with higher biomass tend to yield more. Negative correlations were noted between *Striga* germination score and grain weight (*r* = −0.61), and *Striga* score and fresh weight (*r* = −0.58), indicating that higher *Striga* infestation was associated with reduced yield performance. These relationships underscore the impact of *Striga* on sorghum productivity and the potential of certain traits as indicators of resistance.

### 4.2 Summary of traits and chromosomes with significant SNPs identified via FASTmrMLM and FarmCPU

**FIGURE 2:**
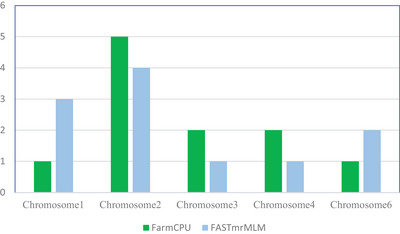
Number of single nucleotide polymorphisms (SNPs) per chromosome for FarmCPU and FASTmrML analysis method.

**FIGURE 3:**
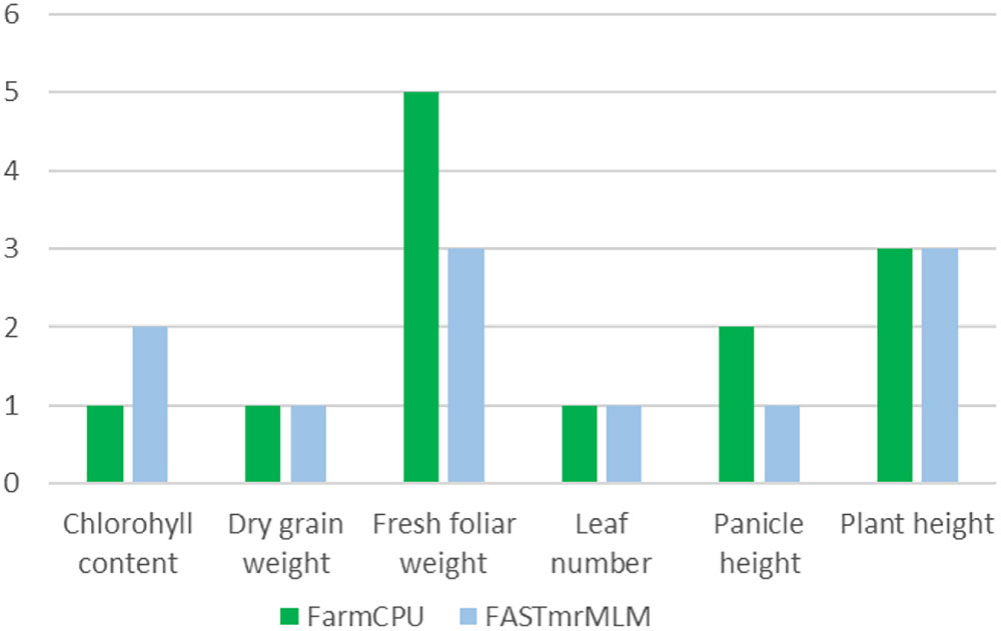
Number of single nucleotide polymorphisms (SNPs) for each trait for FarmCPU and FASTmrMLMeach analysis method.

Chromosomes harboring significant SNPs identified in both FASTmrMLM and FarmCPU models were characterized. Chromosomes 1, 2, 3, 4, and 6 contain SNPs significant for *Striga* tolerance in sorghum, specifically for agronomic‐related traits. Chromosomes 1 (1;3), 2 (5;4), 3 (2;1), 4 (2;1), and 6(1;2) contain SNPs with varying levels of significance for FarmCPU and FASTmrMLM (Figure 2). The agronomic traits measured using FASTmrMLM and FarmCPU showed that the SPNs for plant height (3;1), panicle height (1;2), number of leaves per plant (1;1), field fresh grain weight (3;5), dry grain weight (1;1), and chlorophyll content (2;1) were significant (Figure 3). Fresh field weight contained the highest number of significant SNPs (8). In contrast, the number of leaves per plant and dry grain weight contained the lowest number of SNPs (two) (Figure 3).

### 4.3 Association mapping of agronomic and yield‐related traits of sorghum in response to *Striga* infestation

**FIGURE 4:**
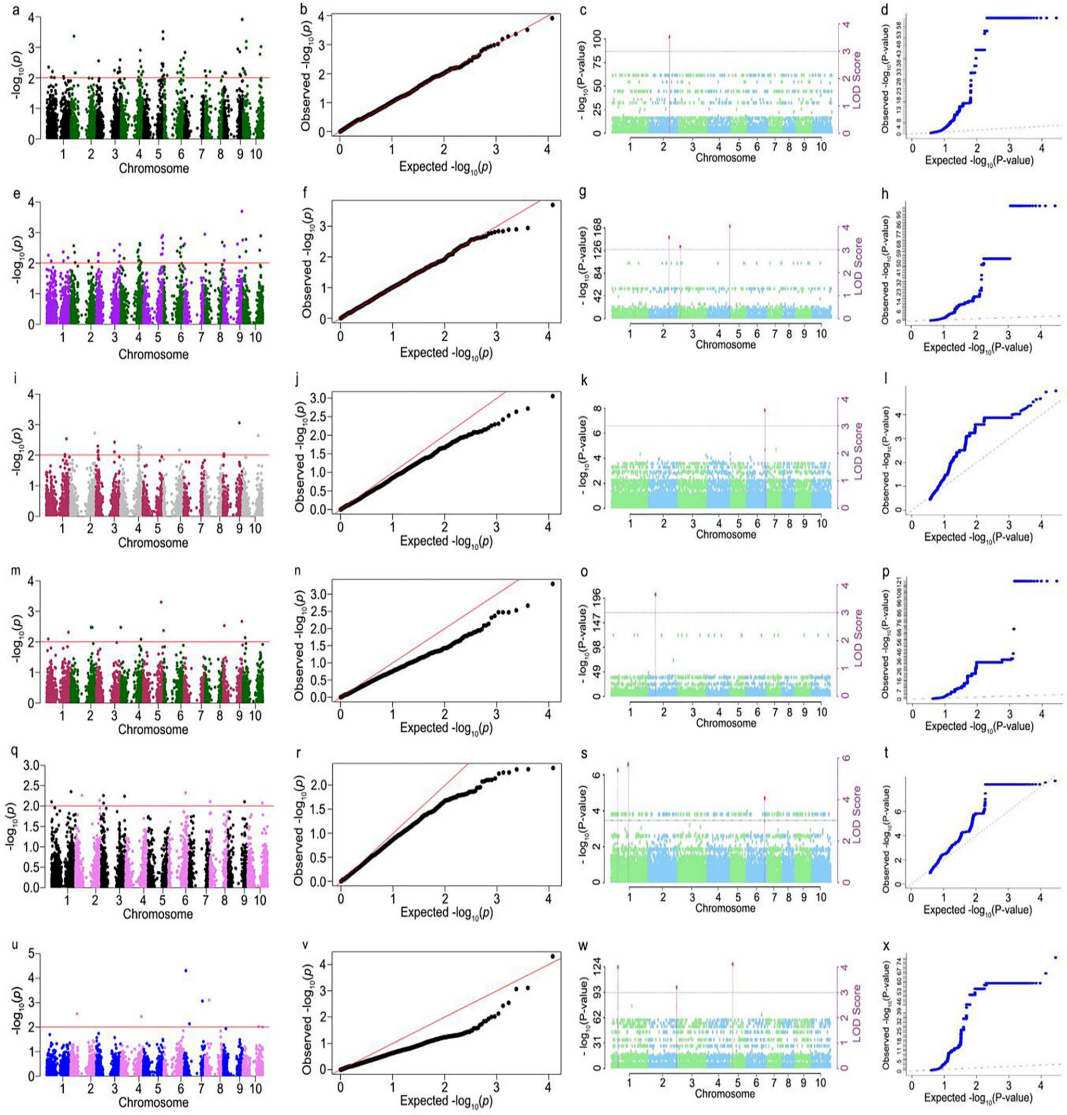
Association analysis results for *Striga* resistance and agronomic traits of 75 sorghum genotypes using the FarmCPU and FASTmrMLM models. (a) Fresh field weight Manhattan plots from association mapping using FarmCPU. (b). Q‐Q plot for fresh field weight using FarmCPU (c). Fresh field weight Manhattan plots using FASTmrMLM. (d) Q‐Q plot for fresh field weight using FASTmrMLM. (e) Dry grain weight Manhattan plots using FarmCPU. (f) Q‐Q plot for dry grain weight using FarmCPU (g). Dry grain weight Manhattan using FASTmrMLM. (h) Q‐Q plot for dry grain weight using FASTmrMLM. (i) Number of leaves Manhattan plot using FASTmrMLM. (j) Q‐Q plot for number of leaves using FarmCPU. (k) Number of leaves Manhattan plots from association mapping using FarmCPU. (l) Q‐Q plot for number of leaves using FASTmrMLM. (m) Panicle height Manhattan plot using FASTmrMLM. (n) Q‐Q plot for panicle height using FarmCPU. (o) Panicle height Manhattan plot using FarmCPU (p). Q‐Q plot for panicle height using FASTmrMLM. (q) Plant height Manhattan pl using FASTmrMLM. (r) Q‐Q plot for plant height using FarmCPU. (s) Plant height Manhattan plot using FASTmrMLM. (t) Q‐Q plot for plant height using FASTmrMLM. (u) Chlorophyll content Manhattan plot using FASTmrMLM. (v) Q‐Q plot for chlorophyll content using FarmCPU. (w) Chlorophyll content Manhattan plot using FASTmrMLM. (x) Q‐Q plot for chlorophyll content using FASTmrMLM. *Note*: The red or solid line denotes the expected distribution of the single nucleotide polymorphisms (SNPs).

Association mapping was performed using the FASTmrMLM model and FarmCPU model to identify loci linked to the evaluated traits. For the FarmCPU model, no significant SNPs were identified at *p *< 0.005; therefore, the Bonferroni threshold was reduced to *p* < 0.001. Multi‐locus random GWAS analysis was performed on agronomic traits using the FASTmrMLM model in the mrMLM package. Association analyses of *Striga* resistance and agronomic traits were performed (Figure 4). The fresh field weight showed that all 10 chromosomes contained significant SNPs above the threshold level using the FarmCPU model (Figure 4a), with chromosomes 2, 3, and 5 having significant SNPs. The Q‐Q plot followed the expected distribution of SNPs (Figure 4b). Analysis using the FASTmrMLM model revealed that chromosomes 2 (2) and 10 (1) contain significant SNPs above the threshold level (Figure 4c). The Q‐Q plots deviated positively from the expected line of the SNPs, with three SNPs at the tail (Figure 4d). The dry grain weight showed that all 10 chromosomes harbor significant SNPs above the threshold level when using the FarmCPU model, with chromosome 9 containing a significant SNP. The Q‐Q plot was below the expected distribution of the SNPs (Figure 4f). Analysis using the FASTmrMLM model revealed that chromosomes 2 (1), 3(1), and 5 (1) contain significant SNPs above the threshold level (Figure 4g). The Q‐Q plots deviated positively from the expected line of the SNPs, with three SNPs at the tail (Figure 4h).

In the FarmCPU model, the number of leaves per plant showed that chromosomes 1, 2, 3, 4, 6, 9, and 10 contain significant SNPs above the threshold level, with chromosome 9 containing a highly significant SNP. The Q‐Q plot was below the expected distribution of the SNPs (Figure 4j). Analysis using FASTmrMLM revealed that chromosome 6 (2) contains significant SNPs above the threshold level (Figure 3k). The Q‐Q plots deviated positively from the expected line of the SNPs, with two SNPs at the tail (Figure 4l). Association analysis using the FarmCPU model for Striga resistance and the number of leaves per plant shows that chromosomes 1, 2, 4, 5, 9, and 10 harbor significant SNPs above the threshold level (Figure 4m), with chromosome 5 containing significant SNP. The Q‐Q plot was below the expected distribution of the SNPs (Figure 4n).

FASTmrMLM analysis revealed that chromosome 2 (2) contains significant SNPs above the threshold level (Figure 4o). The Q‐Q plots deviated positively from the expected line of the SNPs, with three SNPs at the tail (Figure 4p). Using FarmCPU, plant height analysis showed that chromosomes 1, 2, 3, 6, 8, 9, and 10 harbor significant SNPs above the threshold level, with chromosome 1 containing SNPs with high significance (Figure 4q). The Q‐Q plot was below the expected distribution of the SNPs (Figure 4r). Analysis using FASTmrMLM revealed that chromosomes 1 (2) and 6 (1) contain significant SNPs above the threshold level (Figure 3s). The Q‐Q plots deviated positively from the expected line of the SNPs, with three SNPs at the tail (Figure 4t). Chlorophyll content analysis using FarmCPU showed that chromosomes 2, 4, and 7 contain significant SNPs above the threshold level (Figure 4u) with chromosome 7 harboring highly significant SNPs. The Q‐Q plot was below the expected distribution of the SNPs (Figure 4v). Analysis using FASTmrMLM revealed that chromosomes 1 (1), 2 (1), and 5 (1) contain significant SNPs above the threshold level (Figure 3w). The Q‐Q plots deviated positively from the expected line of the SNPs, with three SNPs at the tail (Figure 4x).

## 5 DISCUSSION

The findings show that the way that different sorghum genotypes react to *Striga* infestation varies significantly. Table 2 indicates significant genotypic changes in yield under stress, with considerable variability in fresh weight (CV = 68.08%) and grain weight (CV = 72.25%). Consistent with studies from Ejeta and Gressel (2007), the considerable variation in *Striga* germination scores (CV = 289.33%) further emphasizes varying levels of resistance among genotypes (Ejeta & Gressel, 2007).

Significant effects of treatment (*F* = 595.63, *p* < 0.001), genotype (*F* = 9.37, *p* < 0.001), and their interaction (*F* = 3.51, *p* < 0.001) were confirmed by ANOVA (Table 3). This indicates the severity of the effects of *Striga* as well as the variation in responses between genotypes. Mohamed et al. (2003) showed similar effects of the genotype × *Striga* interaction (Mohamed et al., 2003).

Strong positive correlations between fresh weight and grain weight (*r* = 0.85) and between plant height and fresh weight (*r* = 0.72) were found in the correlation matrix (Figure 1), suggesting that biomass increases yield even in the presence of infestation. In line with other research, *Striga* germination has negative relationships with both grain (*r* = −0.61) and field weight (*r* = −0.58), suggesting that greater infestation lowers productivity (Parker, 2009; Rodenburg et al., 2015). These results highlight the possibility of choosing stable agronomic features in *Striga*‐resistant genotypes for breeding initiatives. Additional molecular research may improve cultivar development and resistance screening.

Although population structure can influence the results of GWAS and increase false connections, GWAS are effective tools for identifying genetic variations related to diverse phenotypes (Abdellaoui et al., 2023). These GWAS models use a kinship matrix to reduce false positives when germplasm accessions share a common ancestor (Wu et al., 2023). Numerous genes conferring essential agronomic traits and *Striga* resistance were identified on the 10 sorghum chromosomes. Additionally, we demonstrated that five of these chromosomes harbor important markers, as indicated by results from FASTmrMLM and FarmCPU analyses. A total of 22 significant SNPs linked to *Striga* resistance were identified in chromosomes 1 (4), 2 (9), 3 (4), 4 (2), and 6 (3) (Figure 2). Upon successful validation, these SNP markers can be utilized for trait introgression and marker‐assisted selection to enhance Striga resistance in sorghum. A varying number of SNPs for agronomic traits corresponding to plant height (4), panicle height (3), number of leaves per plant (2), fresh field grain weight (8), dried grain weight (2), and chlorophyll content (3) were identified (Figure 3).

The greatest number of significant SNPs for *Striga* tolerance was found in fresh field grain weight. A study conducted in Kenya using DArT sequencing classified the genotypes into three primary clusters; all resistance checks, except for N13, fell into the same cluster (Muchira, 2022). The present findings support previous research that identified SNPs linked to *Striga* resistance genes, and this can significantly accelerate the prospects of future breeding projects for Striga resistance (Muchira, 2022)

Numerous studies have examined *Striga* resistance in pearl millet and maize. Laboratory research in Eritrea identified one to three *Striga* resistance QTL in eight genotypes using SSR markers (Tadesse, 2022). Similarly, 28 important SNP markers associated with *Striga* emergence were found on chromosomes 1, 2, 3, 4, 6, and 7 in pearl millet, indicating its potential for trait introgression and MAS to enhance *Striga* resistance (Rouamba et al., 2024). Notably, no SNP overlap was detected in maize between the host plant's *Striga* damage syndrome rating and the number of *Striga*‐emerged plants. However, five SNPs on chromosome 3 showed that the number of *Striga*‐emerged plants overlapped with four QTL found for the host plant's *Striga* damage syndrome rating in previous studies (Gowda et al., 2021; Stanley et al., 2021; Yacoubou et al., 2021), suggesting that this region may carry a significant gene or genes associated with *Striga* resistance.

Furthermore, according to Muchira (2022), “the most consistently *Striga* resistant genotypes across both field and potted artificially infested trials were the F4 generation of F6YQ212 × B35 cross and GBK 045827 wild relative, though not necessarily high yielding as indicated by their HGW values in both trials” (Muchira, 2022). Association mapping was performed using the FASTmrMLM model and FarmCPU model to identify loci linked to the evaluated traits. The FASTmrMLM detected 12 SNPs without adjusting the threshold level. In contrast, FarmCPU failed to detect any significant SNPs before the threshold was adjusted. Ninety‐five SNPs were found to be significant when the Bonferroni threshold was lowered to *p *< 0.001 to identify significant genetic connections (Rosoff et al., 2021). To identify SNPs for follow‐up investigations, Duggal et al. (2008) proposed that a suggestive association threshold should be used, and both nonsignificant and highly significant associations should be considered regions more likely of association (Duggal et al., 2008). Even if a region does not meet the recommended levels, researchers should still consider it significant if it contains promising candidate genes or has biological plausibility. These thresholds are merely guidelines that take into account the interdependence of SNPs (Duggal et al., 2008).

The use of molecular markers in plant breeding is a potential new tool. Under field conditions, they enable the identification and mapping of genes for QTL implicated in polygenic and quantitative resistance, as well as individual and monogenic resistance mechanisms (such as the low stimulant locus). All linkage groups had a high percentage of DArT markers, suggesting that DArT markers are more common than SSRs. According to previous reports (Adu et al., 2024; Li et al., 2022; Mudaki et al., 2023), DArT markers are more likely to map to gene‐rich regions than genomic SSR and AFLP markers (Ali et al., 2016). Analyses of marker‐trait associations have shown that relationships between particular phenotypes and genotypes within a genome may help identify genes that control these traits.

To identify novel sources of *Striga* resistance, Kavuluko et al. (2021) used rhizotrons in a laboratory assay to screen a worldwide sorghum diversity panel and GWAS to uncover the mechanisms of resistance and clarify the genetic loci underlying resistance. They observed QTL overlaps on chromosomes 1, 2, 9, and 10 in both mapped populations. Interestingly, even though the genotype was employed as a donor for pre‐germination resistance, QTL from the IS9830 RIL corresponded to significant SNPs in our GWAS research (Kavuluko et al., 2021). Twenty‐four markers were identified at the threshold of ‐log (*p*) = 4, number of emerged *Striga* plants, number of ears per plant, ear aspect, and grain yield under *Striga* infestation, which increase the level of resistance to *Striga* in the available early maturing tropical maize germplasm. These loci were found on chromosomes 1, 2, 3, 4, and 6. In the present study, 23 SNPs were identified on chromosomes 1, 2, 3, 4, and 6.

In the present study, the parent materials for the intraspecific cross that produced the currently accessible molecular map were selected from the cultivated sorghum gene pool. This molecular map is particularly valuable for real‐world breeding applications, as it provides essential insights into improving sorghum traits. This is because subsequent crossings between the genotypes of cultivated sorghum that are of interest to breeders are likely to have polymorphic segregating DARTSeq markers found within the cultivated gene pool. Sorghum (Haussmann et al., 2002) and several other crops (Al‐Ghamedi et al., 2023; Ali et al., 2016; El‐Rawy & Hassan, 2021; Ren et al., 2003) have exhibited marker clustering with distorted segregation. Most sorghum genetic maps are constructed using wide crosses. A disadvantage of these maps is that the identified loci may only be polymorphic between divergent genotypes. Molecular maps based on crosses involving wild progenitors also have few direct applications in breeding programs, which usually exploit intraspecific variations within cultivated forms. This study offers valuable insights because the population used involves inbred lines and released varieties, which can provide meaningful intraspecific variations among genotypes.

## 6 CONCLUSIONS

Identifying marker‐trait associations is crucial for discovering genomic regions associated with Striga resistance, which is vital for marker‐assisted breeding in sorghum improvement initiatives. Twenty‐two new markers associated with *Striga* resistance were identified. In the future, parental selection, quantitative trait loci analysis, trait introgression, gene pyramiding, and marker‐assisted selection of *Striga* resistance in sorghum breeding programs in Zimbabwe and South Africa, or related agro‐ecologies, will benefit from these novel genetic markers found in current sorghum populations. However, further research is required to validate the markers identified in this study.

## AUTHOR CONTRIBUTIONS

**Wilbert T. Mutezo**: Conceptualization; data curation; formal analysis; investigation; methodology; project administration; software; writing—original draft; writing—review and editing. **Moosa M. Sedibe**: Conceptualization; project administration; resources; supervision; visualization; writing—review and editing. **Justice Norvienyeku**: Data curation; validation; writing—review and editing. **Bingting Lai**: Data curation; software; validation; writing—review and editing.

## CONFLICT OF INTEREST STATEMENT

The authors declare no conflicts of interest.

## Supporting information



Supplementary information on phenotype raw data, met data and SNP data from Dartseq analysis.

## Data Availability

The phenotypic data used in this study, metadata from Dartseq analysis, SNP genetic linkage map positions, SNP physical locations, and SNP MAFs of South African and Zimbabwean inbred accessions are listed in Tables S1–S3 deposited in figshare as shown in the Supporting Information.
