# Supplementary material for: Association mapping for Striga resistance and agronomic‐related traits in sorghum
Source: Plant Genome. 2025 Oct 5;18(4):e70129. doi: 10.1002/tpg2.70129 (PMC12497886; doi:10.1002/tpg2.70129)
Supplement: Supplementary file 1 [file TPG2-18-e70129-s001.docx]

**Supporting information**

Supplementary information on phenotype raw data, met data and SNP data from Dartseq analysis

| **File name** | **Description** |
| --- | --- |
| <https://figshare.com/authors/Wilbert_Mutezo/20145963>  <https://figshare.com/articles/dataset/Agronomic_data_csv/27636297?file=50312286> | Supplementary Table S1. Phenotype data of 75 sorghum genotypes form South Africa and Zimbabwe |
| <https://figshare.com/articles/dataset/metadata_xlsx/27636306?file=50312295> | Supplementary Table S2. Meta data of 75 sorghum genotypes form South Africa and Zimbabwe from SNP analysis using Dartseq |
| <https://figshare.com/articles/dataset/Report_DS24-3019_SNP_mapping_2_csv/27636303?file=50312298> | Supplementary Table S3. Dartseq SNP analysis raw data for 75 sorghum genotypes |
